# The Application of Transient-State Kinetic Isotope Effects to the Resolution of Mechanisms of Enzyme-Catalyzed Reactions

**DOI:** 10.3390/molecules18078230

**Published:** 2013-07-12

**Authors:** Harvey F. Fisher

**Affiliations:** Department of Biochemistry and Molecular Biology, University of Kansas School of Medicine, 3901 Rainbow Blvd, Kansas City, KS 66160, USA; E-Mail: hfisher@kumc.edu

**Keywords:** enzyme mechanism, isotope effects, isotope sensitive step, stopped-flow, quench flow

## Abstract

Much of our understanding of the mechanisms of enzyme-catalyzed reactions is based on steady-state kinetic studies. Experimentally, this approach depends solely on the measurement of rates of free product appearance (d[P]/d*t*), a mechanistically and mathematically complex entity. Despite the ambiguity of this observed parameter, the method’s success is due in part to the elaborate rigorously derived algebraic theory on which it is based. Transient-state kinetics, on the other hand, despite its ability to observe the formation of intermediate steps in real time, has contributed relatively little to the subject due in, some measure, to the lack of such a solid mathematical basis. Here we discuss the current state of existing transient-state theory and the difficulties in its realistic application to experimental data. We describe a basic analytic theory of transient-state kinetic isotope effects in the form of three novel fundamental rules. These rules are adequate to define an extended mechanism, locating the isotope-sensitive step and identifying missing steps from experimental data. We demonstrate the application of these rules to resolved component time courses of the phenylalanine dehydrogenase reaction, extending the previously known reaction by one new prehydride transfer step and two new post hydride transfer steps. We conclude with an assessment of future directions in this area.

## 1. Introduction

Transient-state kinetic isotope effects (tKIEs) are necessarily considered in the context of the general theory of transient-state kinetics, a subject which we will first discuss. At least 98% of our current knowledge of the mechanisms of enzymatic catalysis rests on a basis of steady-state kinetic studies. Experimentally, this approach is limited to the measurement of the reactant concentration dependence on the rates of free product formation. Such steady-state turnover velocities are independent of time and of the nature of the signal measured. A single such measurement provides only a value of the turnover rate under a specified set of conditions. Such a parameter is a complex function whose value involves contributions from every step of the reaction mechanism. A single set of such measurements yields only two parameters: a K_m_ and a V_max_. However, despite the severe limitations of a paucity of phenomena and direct mechanistic relevance, the steady state approach, using pH dependence, product inhibition, isotope effects isotope partitioning, and pulse-chase experiments, is in fact well able to identify the nature and mechanistic location of the internal complexes and even in some cases to characterize the nature of the transition states of their intervening steps! Furthermore, the approach is broadly applicable to nearly all enzymes. This seeming paradox is easily resolved on the following basis: the interpretation of steady-state experimental results rests on the straight-forward rigorously derived algebraic paradigm of W.W. Cleland and its further development by Cleland himself and his many students and followers [1]. It is widely employed and is generally accepted as the authoritative approach to enzyme kinetics. 

Transient-state kinetics, on the other hand, has the decided advantage of providing direct detailed observation of the onset of a rise in the amplitude and subsequent transformation of many optically visible reaction intermediates and does so in real time. Unlike the steady-state approach, whose results are independent of the signal used, transient results are strongly dependent on the signal used and can provide a variety of widely differing time courses, a property which greatly simplifies the constraints necessary for mathematical resolution. Yet, transient state studies have thus far contributed only a minute fraction of our understanding of enzymatic catalysis, usually providing results of *ad hoc* experiments directed at some very specific mechanistic point. Why then, given this clearly superior set of observational phenomena, has the transient state approach made such a minor contribution to the problem? Until now, the transient state approach has lacked any rigorously derived and experimentally applicable mathematical basis for analyzing the phenomenological plethora of data it provides.

The solution of an n-step reaction mechanism poses two quite distinct requirements: (1) sufficient experimental results to provide expressions for independent differential equations plus a conservation equation and (2) an analytic mathematical algorithm capable of organizing the experimental results appropriately into each of these equations. In both the steady-state and the transient-state approaches requirement 1 can be met by collecting the results of a variety of experimental methods. In the steady-state, where each dX/dt term is set to zero, the Cleland approach can solve the resulting set of algebraic equations with relative ease. In the transient-state approach, however where dx_n_/dt is a finite time-dependent function. As discussed below, such an analytic algorithm applicable to realistic enzyme reactions integration of the differential equations has been shown to be mathematically impossible. As a result of the lack of this integrative ability the transient-state kineticist has been forced to use some form of a least-squares empirical fitting routine in its place. Such procedures are beset by a host of problems, imposed by their nature. It is the view of this author that the lack of a valid and applicable analytic algorithm has substantially limited the use of this phenomenonically rich approach, and here we describe a new algorithm which avoids the unattainable requirements of integration of differentials.

## 2. The Basic Theory of Transient-State Kinetics

The general equation for the time course of a given component (C*_i_*) of any reaction of n steps is:

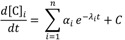
(1)
where α is a time-independent constant relatable to the concentration of the reactant of the ith step at time *t*, *λ* is the phenomenological half-life for that step and C is the concentration of the reactant at *t* = 0.

The solution for a reversible two step reaction:

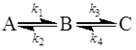
(2)
obtained by integrating the differentials of Equation 1 for components A, B and C is:

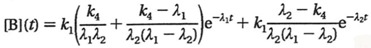
(3)

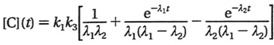
(4)
where 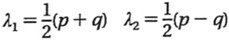 and 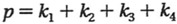, 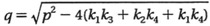 it can be seen from Equation (1) that the λ values represent the slope of their related reaction intermediate at any given point in time while the pre-exponential term provides its corresponding amplitude. Equation (3) and (4) demonstrate the mathematical complexity of λ functions even for such an unreal simplistic two-step reaction. Regrettably, the obviously incorrect assumption that a given λ represents a rate constant for a single step still pervades even the current literature. 

The rigorously derived Equations (3–4) for the two-step reversible case generally constitute the entire formal presentation in otherwise authoritative writings on the subject. This limited treatment of the subject may leave the impression that more complex reaction schemes can also be developed by integration and simultaneous solution of their differential equations. Unfortunately, this assumption is not true for two reasons: (1) it has been proved mathematically that the required closed integral for any mechanism more complex than that of equation (2) does not exist [2]. This fact in itself precludes any further mathematical development along these lines. (2) Bernasconi has pointed out that any two exponentials whose values differ by less than a factor of six are virtually indistinguishable [3]. Thus, the representation of a digitally integrated five-step mechanism may appear to be represented adequately by a simple two exponential expression. 

## 3. Relaxation Approaches

Given the mathematical impossibility of integration of the differential equations for realistic multistep reactions in the transient state, combined with the intractable complexity of the exponential functions for even the two-step reversible case, the prospects for the creation of a rigorously derivable algorithm applicable to the analysis of transient experimental data appeared hopeless. However, in 1963 Manfred Eigen introduced just such a rigorously derived algorithm based on the time dependence of the exponential relaxation of components following the perturbation of a chemical reaction initially at equilibrium [4]. In the form of temperature jump experiments, this analysis did force the requirement of kinetic competence on proposed mechanisms without the necessity of integration of differential equations. This experimental approach also provided access to events in the microsecond time frame, a region not accessible by stopped-flow or quench-flow techniques. The approach was applied successfully only in two cases, each of which provided five distinguishable transient functions, one on the aspartate transaminase reaction [5] by Hammes, and the other by Kirschner (on 3-phosphoglyceraldehyde dehydrogenase [6]). Analysis with Manfred Eigen’s equations led to detailed and generally accepted mechanisms despite the fact that the solutions were admittedly model-dependent and not unique. As noted above, however, most enzyme transient-state experiments revealed only two to three distinguishable exponential steps even for well established eight to ten step reactions. As a result, the T-jump relaxation approach is no longer in general use in resolving enzymatic reactions. 

## 4. Current Transient-State Approaches

Given the demonstrated inability of previously proposed analytical approaches to deal with transient-state experimental data from enzyme reactions studies, the field has diminished to the point where its principal use is limited to *ad hoc* phenomena (frequently supported by arbitrary assumptions of unproved irreversibility for some states and of rapid equilibration of others). The most commonly used current approach involves the numerical integration of stopped-flow or quench-flow spectra followed by single or multi-wavelength empirical fitting of time-courses. Such methods, usually termed “global fitting” routines, are generally based on “singular variable decomposition” (SVD) functions. Such solutions of multi-wavelength transient time courses produce pairs of wavelength and time-dependent vectors. It has not been generally understood that such vectors are combinations of eigenfunctions related to the phenomenological λs of equation (1). As such, they predict behavior of a mechanistic scheme (frequently predicting visibly poor fits to the actual data). Such solutions are beset by two problems: (1) any mechanism of n steps can be fitted with the same general form of equation (1), and (2) for reasons of lack of resolution of closely coupled rate constants as noted by Bernasconi, SVD applications seldom yield more than two to three pairs of usable eigenvalues even for the more than eight step mechanisms that typify most enzyme reactions. K. Johnson has criticized the use of SVD-based functions as lacking validity and in some cases producing incorrect answers [7]. His KinTek program, based on combinations of rate constants producing Venn diagrams, provides additional constraints on the fitting process which substantially improves its effectiveness. Johnson points out that modern computer programs can provide deceptively acceptable “fits” to digitally integrated time courses for assumed mechanisms of greater complexity than are supported by the data. He ascribes the source of this sort of mistaken interpretation to the fact that “standard error analysis grossly underestimates errors” in insufficiently constrained data sets, and points out that such analyses tend to hide the linkages between the multiple complex relationships implicit in equation (1). The KinTek approach provides confidence contour analysis based on sum-square errors on *pairs* of such parameters, providing evidence of such otherwise hidden relationships. He illustrates the ability of the KinTek program to fit published data from well constrained cases and to reveal the lack of reality of a proposed mechanism in an inadequately constrained case despite an apparently acceptable fit. The recently issued 3.0 version of the KinTek program permits the simultaneous solution of multiple data sets, permitting its application to the transient-state KIE approaches proposed here. 

Implicit in Johnson’s discussion is the introduction of a concept new to the field designated as “the FitSpace”. This term recognizes the huge dimensionality of equation (1) of the present paper as analogous to the even larger dimensionality of the process of protein folding now generally designated as the “fold space.” A revised version of the KinTek program has been recently issued which permits the joint solution of multiple independently measured data sets. As such, beyond the substantial increase in mechanistic resolving power provided by such a mathematical constraint, it provides a superior global fitting approach to the direct measurement of transient-state KIEs and provides a rigorous rest of the validity of the fit of any given data set to a proposed mechanism. Yet, the potentially powerful multi-wavelength transient state approach remains largely unused with few fitted time-courses displayed in papers published in the field recently. 

## 5. A New Approach to the Problem

Steady-state kinetic isotope effects (ssKIEs) are by definition independent of both time and the particular signal by which they are measured. tKIEs, on the other hand are strongly dependent on time and on the signal (as well as on the specific mechanistic step to which they apply). As we shall see, these properties provide the basis of a rigorously derived mathematical paradigm for mechanistic resolution of transient-state kinetic data applicable to mechanisms of any length or degree of complexity. However, because of the relative slowing of the isotope substituted reaction, a valid tKIE can be obtained at the limit (t→0) as the reaction time approaches zero. Measured in this manner we have derived three rules of mechanistically dependent behavior which provide at least the sound basis for the mathematical theory whose development we have indicated to be the necessary ingredient for useful interpretation of transient-state experimental data. 

## 6. Transient-State Experimental Methods

**Figure 1:**
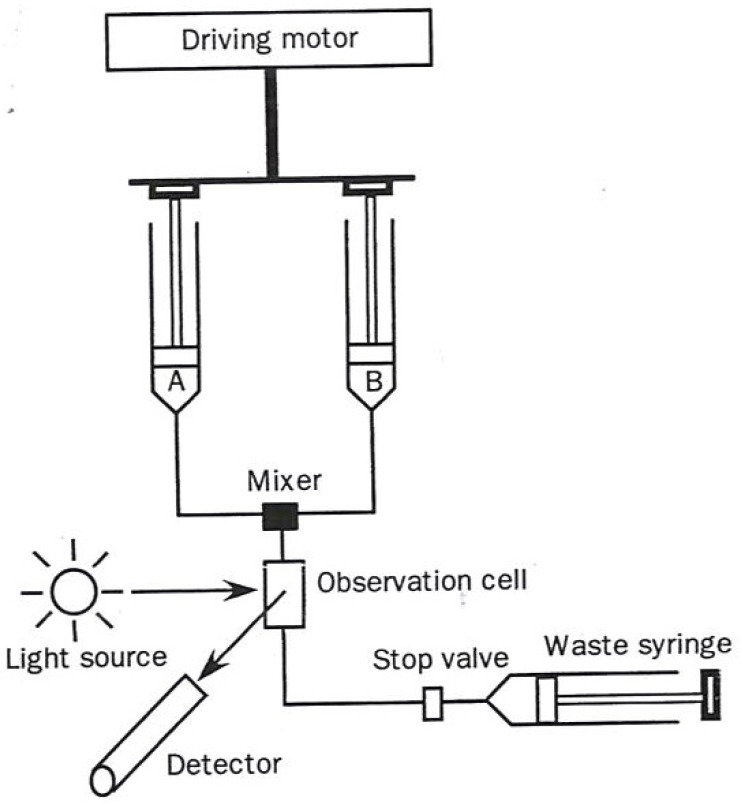
Diagram of a stopped flow apparatus used in kinetic measurements. The figure was adapted from one available on p. 221 [1].

**Figure 2:**
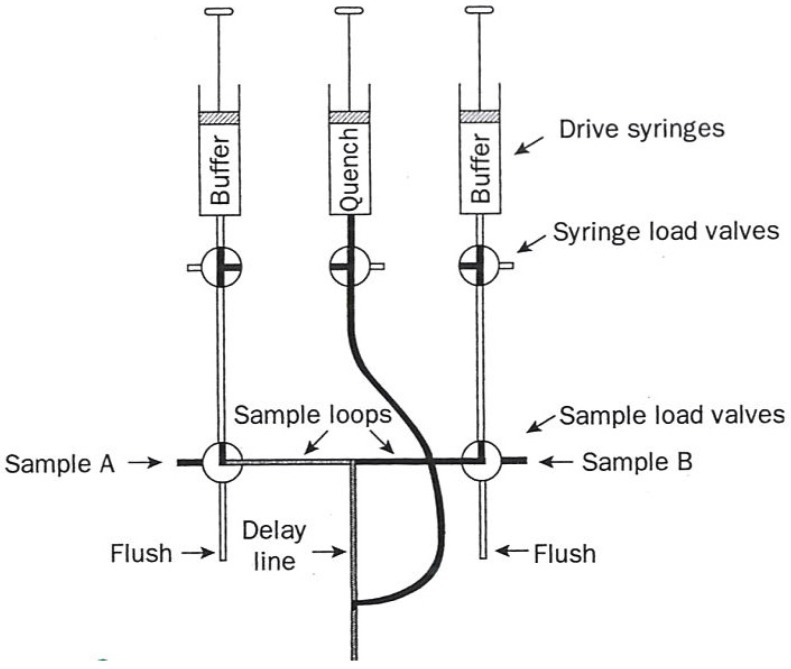
Diagram of a flow quench apparatus used in kinetic measurements. The figure was adapted from one available on p. 221 [1].

The two most commonly used transient-state techniques are (1) Stopped flow (diagrammed in Figure 1), and (2) flow quench (diagrammed in Figure 2).

In the stopped flow approach a solution in one syringe containing enzyme alone and one containing substrate alone in a second syringe are simultaneously driven through a rapid mixer resulting in a brief temporary continuous flow reactive solution, passing through an optical detecting device. At the point in time when the forward movement of the two syringes encounters a mechanical stop, the partially reacted mixed solution begins its time-dependent reaction. The length of time in which the mixed solution must travel to the sensing device (usually 1–3 milliseconds) is designated as the “dead time”.

In quench flow, the reaction mixture no longer enters a third syringe but instead a third (quench) solution is added to the path leading to a collection vessel at some fixed distance after mixing. This solution involves a rapid-reaction-stopping agent such as acid, hydrophobes, or cryogens. Less than 15 µL of solution are required per reaction. 

Each aliquot so obtained is then resolved into its specific chemical entities which are then quantified by their appropriate analytical techniques such as HPLC.

## 7. The Rules of Transient-State Kinetic Isotope Effects

We define a transient state kinetic isotope effect on a given component, x, at any stated point in time as:

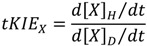
(5)
where “h” and “d” designate the protio-and deuterio-substituted forms of each “x” species.

### 7.1. The First Rule of Transient-State KIEs

**Scheme 1:**
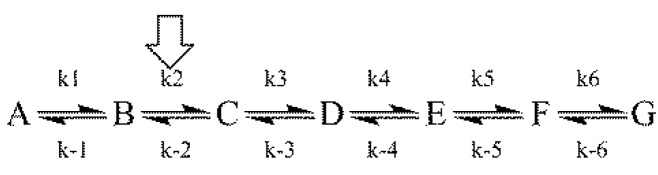
An assumed reaction mechanism.

**Figure 3:**
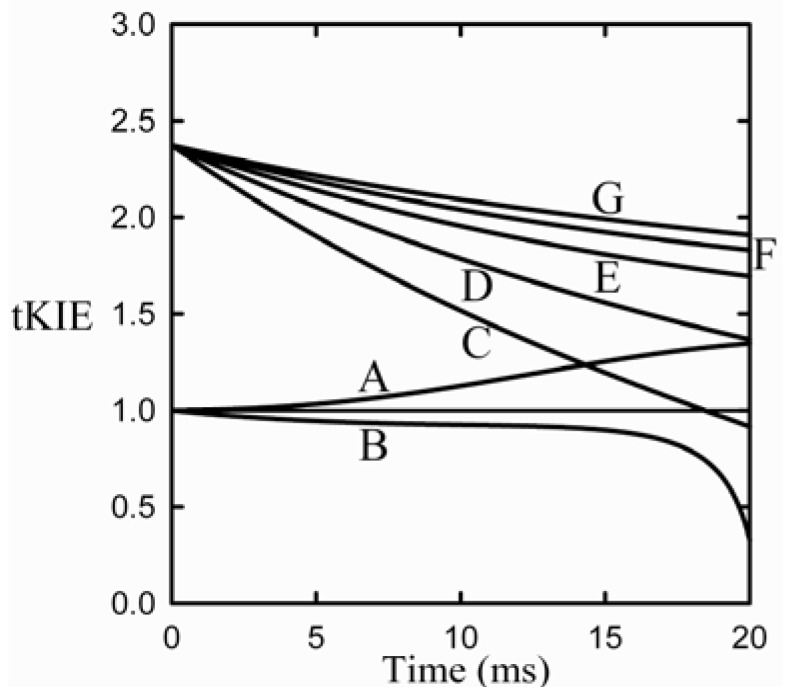
Application of the first rule to an assumed reaction mechanism. This example assumes an intrinsic KIE = 2.3, and that the iss is step 2 [9].

This rule states that for any *post* single site substituted reaction:

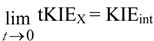
(6)
and that for any *pre* isotope sensitive step in such a reaction:

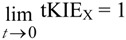
(7)
Both aspects of the First Rule to an assumed linear reaction sequence using a simulation based on the numerical integration of the equations derived from Scheme 1 [where the arrow designates the isotope-sensitive step (iss)] are shown in Figure 3 [8]. 

It can be seen from the figure that post-iss tKIEs for the reactions of components C through G all converge to a single point which has the precise value of the assumed intrinsic KIE of 2.3. On the other hand, those of the pre-iss steps, A and B, converge at a value of unity. A variety of such simulations assuming widely varying values of both rate constants and KIE_int_ revealed no deviations from the predictions of the First Rule. 

### 7.2. The Second Rule of Transient-State KIEs

**Figure 4:**
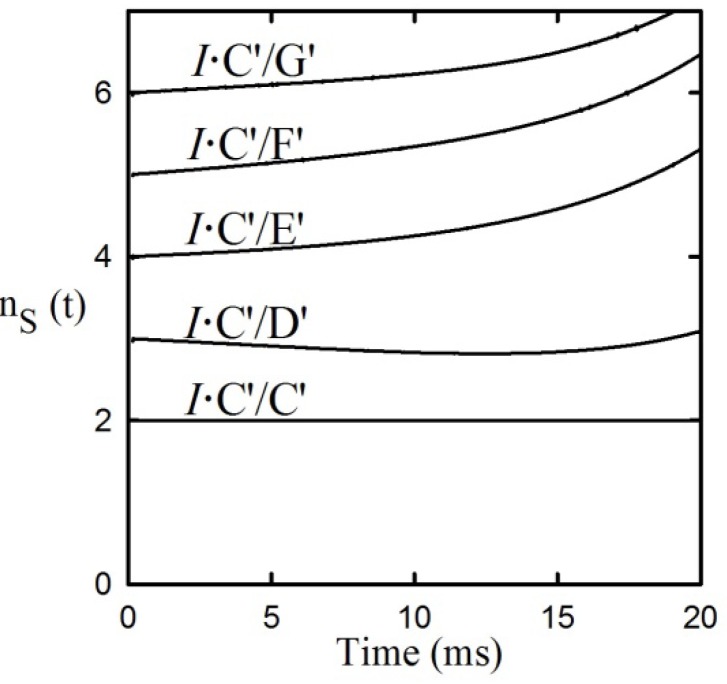
The second rule time course of the first derivatives of the tKIEs applied to the mechanism portrayed in Scheme 1. *I* is set to a value of 2 here as required by the location of the iss in this example.

It can be seen from Figure 3 that the tKIEs of the various post iss steps decrease at rates in order of their step number. Exploration of this phenomenon, first by simulation and later by rigorous derivation resulted in the rule:

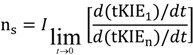
(8)
where n_s_ is the actual mechanistic step number, subscript 1 designates the first post iss, and subscript n indicates the number of successive steps and I is an integral number equal to the number of steps including and preceding the iss [8]. The validity of this Rule is demonstrated by the results of the application of equation (8) to the component tKIE time courses of Figure 3 shown in Figure 4, which show t→o intercepts of successive perfect integers. 

The notion that equations based on kinetic time courses can provide absolute integers corresponding to those of mechanistic step numbers appears to be counterintuitive. It can be seen, however, from equation 1 that at the introduction of each successive step a new term having the form of αe^−λt^ is added to the equation. The process used for determining the values of the limits of t→0 in equation (8) involves application of L’Hȏpital’s rule. The nature of this rule involves successive differentiation. Each such operation yields the indeterminate value of 0/0 until their number reaches the value of “n” for that particular reaction step. Since neither the number of steps in a reaction nor the number of times a term is differentiated can be fractional, equation (8) can yield only integral values. The validity of this Rule is demonstrated by its application to the simulated data of Figure 3 whose results are shown in Figure 4. 

## 8. Application of the First and Second Rules of Transient-State Kinetic Isotope Effects to an Enzymatic Mechanism

The system chosen for this task is the reaction catalyzed by the pyridine nucleotide linked phenylalanine dehydrogenase (PheDH) catalyzed reaction. The stoichiometry of this class of enzyme reactions is:

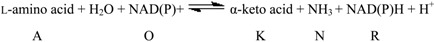
(9)
The bold letters shown below each species will be used throughout this report. A is L-amino acid, K is α-keto acid, N is ammonia, O is NADP+ (oxidized coenzyme), and R is NAD(P)H (reduced coenzyme). The basic chemical mechanism has been shown to be that of equation (10) (the boldface abbreviations in these two equations will be used throughout the following discussion). Other members of the class have similar active site structures, and their reactions involve the same sequences of complexes [9].


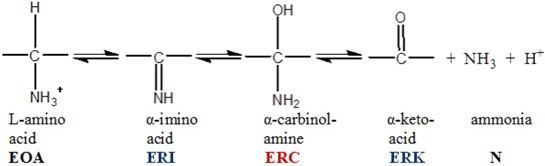
(10)

**Figure 5:**
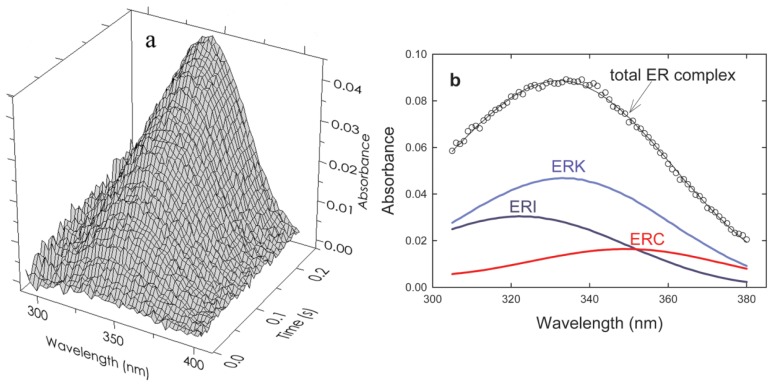
(**a**) Time and wavelength dependence of the optical absorbance of the phenylalanine dehydrogenase catalyzed reaction. The array of 121,600 experimental points has been parsed here for clarity. The final reactant concentrations after mixing were 45 µM PheDH, 1.0 mM NAD and 1.0 mM h- or d-l-Phe at pH 8.0. (**b**) The resolution of a typical spectrum at a single point in time (80 ms) indicated by the open circles. Beer’s law compliance is obtained as shown by the solid line through the open circles representing the sum of the absorbances of all components.

**Figure 6:**
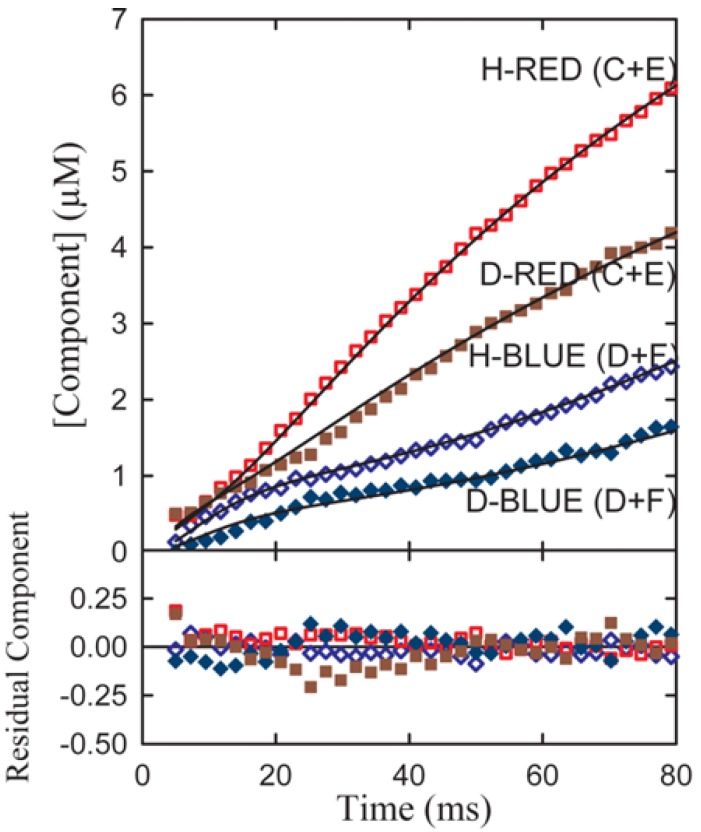
A simultaneous fit of all four components to both the h(protio) and d(deuterio) component time course of the resolved date of Figure 3 where C and D follow the sum of the red and blue components respectively. Red-shifted components are indicated by diamond shaped symbols and blue-shifted components by circles. Open symbols denote h-substituted components; closed symbols indicate d-substituted components [10].

**Figure 7:**
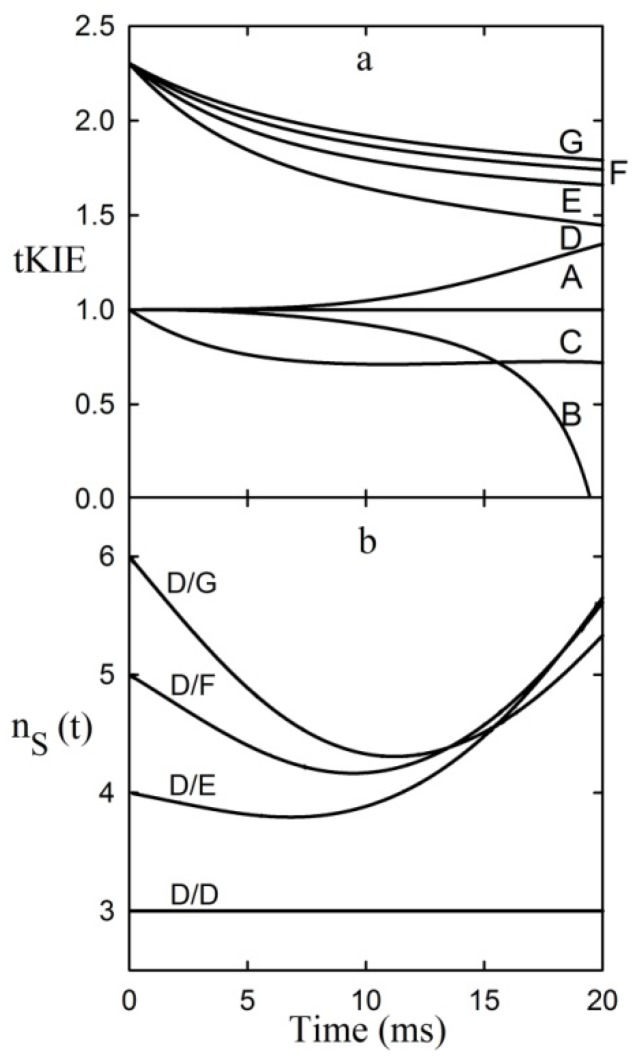
Application of the First and Second Rules to the PheDH reaction. (**a**) Application of the First Rule-time courses of tKIEs of the reaction components assuming the six-step mechanism of Equation (11). (**b**) Application of the Second Rule using the n_s_ values calculated from the fitted curves of panel a.


The coloring in equation (10) and subsequently in Figure 5b indicates the direction of the shift in the 340 nm peak of free R(NADPH) as it occurs in the various complexes. The results of a typical experiment (absorbance *vs* wavelength *vs* time) are shown in Figure 5a. The deconvolution of an individual “time slice” of this array into specific components is shown in Figure 5b. Application of the First and Second rules to that data are shown in Figure 5, Figure 6, Figure 7 (from reference [10]). 

Experimental transient time courses for the phenylalanine dehydrogenase reaction for both α-h and α-d phenylalanine assembled from series of resolutions typified in Figure 5b are shown in Figure 6.

Application of the First and Second rules to the data shows in Figure 5 failed to provide integral n_s_ values for 3,4 and 5 step applications of the Second Rule. However, application of the integral n_S_ values for both pre- and post-iss step numbers to the required expansion of the central portion of the reaction from 4 to 7 steps including 2 pre-iss steps as shown in equation (11) did satisfy the rule, as shown in Figure 7, providing the mandatory integral values shown in Figure 7b. It can be seen that each of the two red and two blue signals shown by the data in Figure 6a have now been split into four distinguishable component time courses and that the iss has been moved to step 3. [The trended misfits of theory to data in the 25–35 ms region may be due either to errors in spectroscopic resolution or to a yet unrecognized mechanistic feature clearly evident form the expanded residual plot below panel a].

The application of these rules to the phenylalanine dehydrogenase catalyzed reaction [10] represents the first model-independent, assumption free and multi-step applicable approach to the mechanistic resolution of transient-state enzyme kinetic data. As such, it represents the current state of the art. Nevertheless, close examination of the residual error panel reveals several small but significant trended deviant areas suggesting further yet undiscovered mechanistic complexity.

This successful application of the Second Rule to the mechanism of the phenylalanine dehydrogenase provides a particularly severe test of the rule’s mechanistic resolving power. Unlike the results of stopped flow experiments on L-glutamate dehydrogenase reactions, which yielded four to five spectroscopically distinguishable intermediate species, the corresponding L-phenylalanine dehydrogenase experiments yielded only two such signals each of which corresponded to at least two components. Nevertheless, application of the Second Rule, using the complexity of the signal time courses, was able to distinguish them into kinetically competent entities.

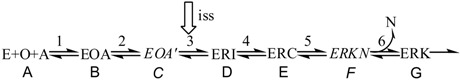
(11)

Palfey and Fagan [11] explored the scope of the applicability of the Second Rule to various mechanistic patterns and identified a number of limits to that scope. This rule has been proven valid for linear mechanisms of any length and for those containing closed loops leading to a single set of products. In such cases, however, n_s_ will number the steps for the shortest pathway even in cases where a longer pathway may be faster. The rule fails only in the case where the proposed mechanism is inadequate or where an adequate mechanism contains an open loop. In such a case n_s_ will result in a numerical average of the two separate individual values; in general, such a value will not be a simple integer.

Quite aside from its basic role in applying the tKIE rules as discussed above, the measurement of iss-deuteron-substituted reactions (such as those shown in Figure 7) serve a second very useful additional function; by providing a second set of component time course they double the number of constraints that can be applied to the resolution of transient state data in terms of the set of differential equations that express a proposed mechanism.

## 9. The Third Rule of Transient-State KIEs

This rule states that for multi-step isotope substituted reactions:

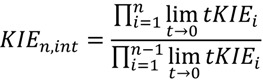
(12)

Simply stated, in such a sequence the tKIE observed for a given species at t→0 is the algebraic product of all the intrinsic KIEs for the forward reaction steps preceding the formation of the product of the nth step. Palfey and Fagan have provided a rigorous analytic derivation for this rule which is applicable principally to solvent isotope (D_2_O) studies [11]. Sandro Ghisla has verified the validity of this rule for a flavin-enzyme catalyzed reaction (personal communication). Schneck* et al.* have applied the third rule in their solvent KIE study of a cysteine protease [12].

## 10. Scope of Applicability of the tKIE Rules

While the experimental work in support of the theory described above has been based on the multi-wavelength stopped-flow approach described elsewhere [13], the theory is equally applicable to the quench flow technique which applies chemical analysis to released products at various points in the transient-state time range. As such, it does not require chromophoric or fluorescent substrates, greatly expanding the range of enzyme systems from which tKIE_int_ values can be obtained. From this feature two points follow: (1) While only the D/H mass ratio of 2 is sufficient for the determination of a KIE by direct comparison of reaction rates, it has been shown that ratio-mass spectrometry of aliquots of steady-state enzyme reactions is sufficiently accurate to permit the measurement of KIEs of such stable isotopes as ^13^C, ^15^N, and ^18^O [1]. Weiss* et al.* used a combination of steady-state hydrogen and nitrogen KIEs effects to establish the basic sequence of chemical entities common to all pyridine nucleotide-linked amino acid dehydrogenases as shown in equation (11) [14]. (2) Such a multinuclear approach, which requires the use of the quench flow technique, should free the three rules of tKIEs from the limitation of applicability to C-H bond-breaking mechanisms alone, and permit its potential use in exploring the mechanisms of enzymatic mechanisms in general. To our knowledge, this approach has not yet been attempted. 

The three approaches to the kinetic investigation of mechanisms of enzymatic catalysis, steady state, stopped flow and quench flow should not be considered as either competitive or even alternative methods; each has the ability to provide unique contributions to solving specific aspects of the problem and each has its limitations and blind spots. Thus, the proper investigation of an enzymatic reaction should involve the application of all three approaches.

Given the findings of K. Johnson of the inadequacy of Chi^2^ as a criterion of kinetic competence in refernece [7] it is also clear that the limited body of rigorously derived and experimentally applicable analytic theory available at present is inadequate to take full advantage of the phenomenological cornucopia provided by transient state studies. Further advances in this area must be pursued. 
